# *CCNF* mutations in amyotrophic lateral sclerosis and frontotemporal dementia

**DOI:** 10.1038/ncomms11253

**Published:** 2016-04-15

**Authors:** Kelly L. Williams, Simon Topp, Shu Yang, Bradley Smith, Jennifer A. Fifita, Sadaf T. Warraich, Katharine Y. Zhang, Natalie Farrawell, Caroline Vance, Xun Hu, Alessandra Chesi, Claire S. Leblond, Albert Lee, Stephanie L. Rayner, Vinod Sundaramoorthy, Carol Dobson-Stone, Mark P. Molloy, Marka van Blitterswijk, Dennis W. Dickson, Ronald C. Petersen, Neill R. Graff-Radford, Bradley F. Boeve, Melissa E. Murray, Cyril Pottier, Emily Don, Claire Winnick, Emily P. McCann, Alison Hogan, Hussein Daoud, Annie Levert, Patrick A. Dion, Jun Mitsui, Hiroyuki Ishiura, Yuji Takahashi, Jun Goto, Jason Kost, Cinzia Gellera, Athina Soragia Gkazi, Jack Miller, Joanne Stockton, William S. Brooks, Karyn Boundy, Meraida Polak, José Luis Muñoz-Blanco, Jesús Esteban-Pérez, Alberto Rábano, Orla Hardiman, Karen E. Morrison, Nicola Ticozzi, Vincenzo Silani, Jacqueline de Belleroche, Jonathan D. Glass, John B. J. Kwok, Gilles J. Guillemin, Roger S. Chung, Shoji Tsuji, Robert H. Brown, Alberto García-Redondo, Rosa Rademakers, John E. Landers, Aaron D. Gitler, Guy A. Rouleau, Nicholas J. Cole, Justin J. Yerbury, Julie D. Atkin, Christopher E. Shaw, Garth A. Nicholson, Ian P. Blair

**Affiliations:** 1 Department of Biomedical Sciences, Faculty of Medicine and Health Sciences, Macquarie University, Sydney, New South Wales 2109, Australia; 2 Northcott Neuroscience Laboratory, ANZAC Research Institute, Sydney, New South Wales 2139, Australia; 3 Sydney Medical School, University of Sydney, Sydney, New South Wales 2006, Australia; 4 Medical Research Council Centre for Neurodegeneration Research, Department of Clinical Neuroscience, Institute of Psychiatry, King's College London, London SE5 8AF, UK; 5 Illawarra Health and Medical Research Institute, School of Biological Sciences, University of Wollongong, Wollongong, New South Wales 2522, Australia; 6 Department of Genetics, Stanford University School of Medicine, Stanford, California 94305, USA; 7 Montreal Neurological Institute and Hospital, Department of Neurology and Neurosurgery, McGill University, Montreal, Québec, Canada H3A 2B4; 8 Pathology and Cellular Biology Department, Montreal University, Montreal, QC H3T 1J4 Québec, Canada; 9 Australian Proteome Analysis Facility, Macquarie University, Sydney, New South Wales 2109, Australia; 10 Department of Biochemistry, La Trobe University, Melbourne, Victoria 3086, Australia; 11 Neuroscience Research Australia, Randwick, Sydney, New South Wales 2031, Australia; 12 School of Medical Sciences, University of New South Wales, Kensington, Sydney, New South Wales 2052, Australia; 13 Department of Neuroscience, Mayo Clinic Florida, Jacksonville, Florida 32224, USA; 14 Department of Neurology, Mayo Clinic Rochester, Rochester, Minneapolis 55905, USA; 15 Department of Neurology, Mayo Clinic Florida, Jacksonville, Florida, 32224, USA; 16 Medical Genome Center, The University of Tokyo Hospital, The University of Tokyo, Tokyo 113-8655, Japan; 17 Worcester Polytechnic Institute, Worcester, Massachusetts 01609, USA; 18 Department of Neurology, University of Massachusetts Medical School, Worcester, Massachusetts 01605, USA; 19 Unit of Genetics of Neurodegenerative and Metabolic Diseases, Fondazione IRCCS Istituto Neurologico ‘Carlo Besta', 20133 Milan, Italy; 20 School of Clinical and Experimental Medicine, College of Medical and Dental Sciences, University of Birmingham, Birmingham B15 2TT, UK; 21 The Queen Elizabeth Hospital, Woodville South, South Australia 5011, Australia; 22 Department of Neurology, Emory University, Atlanta, Georgia 30322, USA; 23 Unidad de ELA, Instituto de Investigación Hospital Gregorio Marañón de Madrid, Sermas 28007, Spain; 24 Unidad de ELA, Instituto de Investigación Hospital 12 de Octubre de Madrid, Sermas 28041, Spain; 25 Centro de Investigación Biomédica en Red de Enfermedades Raras (CIBERER U-723), Madrid 28029, Spain; 26 Banco de Tejidos, Centro Alzheimer—Fundación Reina Sofia, Fundación CIEN, Madrid 28071, Spain; 27 Academic Unit of Neurology, Trinity Biomedical Sciences Institute, Trinity College Dublin, Dublin 2, Republic of Ireland; 28 Queen Elizabeth Hospital, University Hospitals Birmingham NHS Foundation Trust, Birmingham B15 2TH, UK; 29 Faculty of Medicine, University of Southampton, Southampton SO17 1BJ, UK; 30 Department of Neurology and Laboratory of Neuroscience, IRCCS Istituto Auxologico Italiano, 20149 Milan, Italy; 31 Department of Pathophysiology and Transplantation, ‘Dino Ferrari' Center—Università degli Studi di Milano, 20122 Milan, Italy; 32 Neurogenetics Group, Division of Brain Sciences, Imperial College London, Hammersmith Hospital Campus, London W12 0NN, UK; 33 Department of Neurology, Graduate School of Medicine, The University of Tokyo, Tokyo 113-8655, Japan; 34 Molecular Medicine Laboratory, Concord Hospital, New South Wales 2139, Australia

## Abstract

Amyotrophic lateral sclerosis (ALS) and frontotemporal dementia (FTD) are overlapping, fatal neurodegenerative disorders in which the molecular and pathogenic basis remains poorly understood. Ubiquitinated protein aggregates, of which TDP-43 is a major component, are a characteristic pathological feature of most ALS and FTD patients. Here we use genome-wide linkage analysis in a large ALS/FTD kindred to identify a novel disease locus on chromosome 16p13.3. Whole-exome sequencing identified a *CCNF* missense mutation at this locus. Interrogation of international cohorts identified additional novel *CCNF* variants in familial and sporadic ALS and FTD. Enrichment of rare protein-altering *CCNF* variants was evident in a large sporadic ALS replication cohort. *CCNF* encodes cyclin F, a component of an E3 ubiquitin–protein ligase complex (SCF^Cyclin F^). Expression of mutant *CCNF* in neuronal cells caused abnormal ubiquitination and accumulation of ubiquitinated proteins, including TDP-43 and a SCF^Cyclin F^ substrate. This implicates common mechanisms, linked to protein homeostasis, underlying neuronal degeneration.

## Abstract (web summary)

Ian Blair and colleagues use genome-wide linkage analysis and whole exome sequencing to identify mutations in the *CCNF* gene in large cohorts of amyotrophic lateral sclerosis and frontotemporal dementia patients. In addition to validating the mutations in international cohorts, the authors also show that mutant *CCNF* gene product affects ubiquitination and protein degradation in cultured cells.

## Graphical abstract

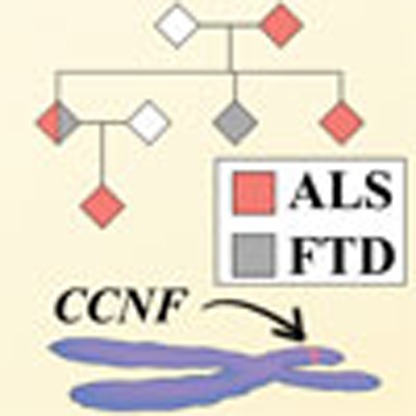

Amyotrophic lateral sclerosis (ALS) is a late-onset fatal disorder characterized by the progressive degeneration of upper and lower motor neurons. Approximately 10% of ALS cases have a positive family history (familial ALS) and appear clinically indistinguishable from sporadic ALS cases. Mutations in several genes including *SOD1*, *TARDBP*, *FUS*, *UBQLN2*, *PFN1*, *OPTN*, *VCP*, *MATR3*, *TUBA4A* and *C9ORF72* account for approximately two-thirds of familial ALS and 5% of sporadic ALS cases^1^,^2^,^3^,^4^,^5^,^6^,^7^,^8^,^9^,^10^,^11^. Other ALS families are not linked to known loci and the cause of most sporadic ALS also remains unknown. Upto 15% of ALS patients are also diagnosed with frontotemporal dementia (FTD) and segregation of both ALS and FTD may be seen within families, particularly those with mutations in *C9ORF72* (refs 7, 8).

The aetiology of ALS remains poorly understood but recent ALS gene discoveries are providing insight into pathological disease mechanisms and are critical to the development of *in vitro* and *in vivo* models to study pathogenesis. Coupling linkage analysis with next-generation sequencing provides a powerful approach for the identification of novel ALS genes. Here we use this approach to discover a pathogenic *CCNF* mutation in a large ALS-FTD family. Additional *CCNF* mutations were identified in diverse international familial ALS and FTD cohorts. Significant enrichment of novel and rare protein-altering variants in *CCNF* was observed in a replication cohort of sporadic ALS cases relative to controls. A hallmark pathological feature of most ALS and FTD cases is the presence of abnormally ubiquitinated proteins, particularly TDP-43, in neuronal cytoplasmic inclusions^12^. We demonstrated that mutant cyclin F, encoded by *CCNF*, resulted in abnormal ubiquitination and accumulation of ubiquitinated proteins, including TDP-43.

## Results

### Linkage mapping implicates a new ALS locus on chromosome 16

**Figure 1:**
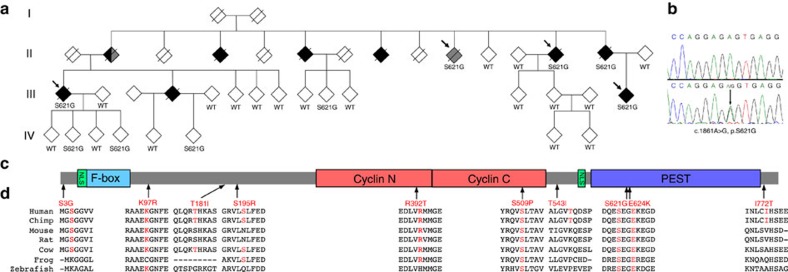
*CCNF* mutations in ALS-FTD identified following genetic linkage analysis and exome sequencing. (**a**) Pedigree of family FALS10. All family members from whom DNA was available for genotyping are indicated by either wild type (WT) for *CCNF*, or by mutation in *CCNF* (‘S621G'). Individuals with ALS are represented by a black-filled symbol, individuals with FTD by a grey-filled symbol. Arrows indicate samples used for exome sequencing. (**b**) Sequence traces of WT and c.1861A>G mutation identified in family FALS10. (**c**) Diagrammatic representation of cyclin F protein and the location of novel mutations identified in this study. Cyclin F contains three functional modules within its protein structure. The F-box domain forms a ‘pseudocatalytic' module, the two cyclin domains form the substrate recruitment module and the C terminus contains both a nuclear localization signal (NLS) and a PEST sequence (short stretch of amino acids enriched in proline, glutamic acid, serine and threonine) that form the regulatory module^16^. (**d**) Multiple sequence alignment of cyclin F across species showing evolutionary conservation of the substituted amino-acid residues (indicated by arrows). Sequences include NP_001752.2 (human), NP_001252844.1 (chimpanzee), NP_031660.3 (mouse), NP_001093944.1 (rat), NP_001092340.1 (cow), NP_001079901.1 (frog) and NP_996931.1 (zebrafish).

To identify novel ALS loci, we performed two independent genome-wide scans for linkage in an Australian multi-generational family (FALS10, historically of UK ancestry) comprising 10 individuals with ALS and/or FTD (Fig. 1a). The family displays autosomal dominant inheritance of disease and mutations in all known ALS genes were excluded. The first genome-wide scan for linkage, which used 10 K Affymetrix single-nucleotide polymorphism (SNP) arrays, implicated candidate loci with maximum multipoint LOD scores >1.9 but <3, on chromosomes 11p15.4-p14.3, 16p13.3-p12.3 and 20p13-p12.2, respectively (Supplementary Fig. 1b). The second genome-wide scan for linkage, which used 539 microsatellite markers, generated LOD scores >3.0 at a single candidate region on chromosome 16 (maximum genome-wide two-point LOD score of 3.16, *θ*=0, at D16S3065) that overlapped the chromosome 16 interval identified by the initial, SNP-based, linkage scan (Supplementary Fig. 1a). Fine mapping and recombinant haplotype analysis using a dense coverage of microsatellite markers spanning the chromosome 16 interval refined the minimal candidate region to 7.5 Mb on chromosome 16p13.3 flanked by D16S521 and D16S418, with a maximum two-point LOD score of 3.24, *θ*=0, at D16S3082 (Supplementary Table 1). Multipoint linkage analysis of the 7.5 Mb disease haplotype indicated LOD scores >3 across the entire linked disease interval (Supplementary Fig. 1c). In summary, unbiased genome-wide linkage analysis in family FALS10 identified a single novel familial ALS/FTD locus on chromosome 16p13.3.

### A missense mutation in *CCNF* in a large ALS/FTD family

Four family members with ALS or FTD (FALS10, individuals II:10, II:13, III:1 and III:15) were chosen for whole-exome sequencing. The mean read depth for these patients was × 119.3, with an average of 6.01 × 10^9^ base pairs sequenced per individual. To identify candidate mutations, exome sequence variants were annotated and filtered (Supplementary Table 2) using the following criteria: the variant was present in four affected family members, resulted in altered amino-acid sequence, and was absent from public SNP databases including dbSNP137, the 1000 Genomes Project (>0.001 frequency, October 2011 release), the NHLBI Exome Sequencing Project (ESP) exome variant server (6,503 sequenced human exomes), and ExAC database (>0.001 frequency). Of the two variants that remained following filtering, one was located within the linked region on chromosome 16p13.3 and the other lies in a region on chromosome 22 that was excluded by linkage analysis (LOD scores <−2 for multiple flanking markers). The variant in the linked region lies in the *CCNF* gene, leads to an A to G substitution at position 1,861 of the coding DNA (c.1861A>G) and results in an amino-acid substitution of serine with glycine at codon 621 at the protein level (p.S621G). Sanger sequencing of 29 family members demonstrated segregation of the mutation in all affected family members for whom DNA was available (three ALS and one FTD). We also genotyped the offspring of other ALS patients for whom DNA was unavailable (deceased), and demonstrated segregation of the mutation in a further three patients (that is, obligate carriers, Fig. 1a,b). As such, we have shown segregation of the *CCNF* c.1861A>G mutation in seven ALS patients from family FALS10. The mutation was present in four at-risk family members (three <40 years, one <60 years). This variant was absent from 1,831 control individuals recruited from the same population. This variant is present in the ExAC database as a singleton (MAF=8.629 × 10^−6^). It is important to note that the ExAC database also includes other reported ALS/FTD mutations as singletons (including *SOD1*, *FUS* and *GRN*). Here unbiased whole-exome sequencing in family FALS10 identified two novel missense variants as candidate pathogenic mutations, one of which lies within the novel linked locus on chromosome 16p13.3 and segregated with disease.

### *CCNF* variants in ALS/FTD from diverse geographic populations

To determine whether mutations in *CCNF* are present in other ALS and FTD patients, we used targeted sequencing, whole-exome sequencing or whole-genome sequencing in the following discovery cohorts: index cases from 75 Australian ALS families, 159 UK ALS families, 108 USA ALS families, 100 Canadian ALS families, 99 Italian ALS families, 32 Japanese ALS families, 30 Spanish ALS families, 16 Irish ALS families, 283 Japanese sporadic ALS cases, 168 French-Canadian sporadic ALS cases, 26 USA sporadic ALS cases, 49 USA ALS trios, 99 USA FTD (FTLD-TDP) cases, 43 Australian FTD families and 29 Australian sporadic FTD cases. In familial ALS and/or FTD patients, we identified five additional novel missense mutations in *CCNF* (Table 1). In sporadic ALS and/or FTD patients, we found 19 protein-altering variants in *CCNF* (missense, nonsense and frameshift), including seven novel variants (Supplementary Table 4). Sanger sequencing confirmed all novel *CCNF* variants. Most of the novel variants substitute amino acids that are highly conserved across species (Fig. 1d). All variants were examined in large control cohorts and data sets including: dbSNP; NHLBI ESP Exome Variant Server, 1000 Genomes Project, ExAC, the Japanese Human Genetic Variation Database and 967 Australian control exomes (of European ancestry). Selected variants were also tested in population-specific control cohorts from the UK, USA, Canada and Japan. None of the familial mutations or novel sporadic variants were present in genotyped controls and were absent from the public databases (Table 1). The average age of disease onset in familial ALS cases with a *CCNF* mutation is 55.3±8.0 (Supplementary Table 3). Detailed clinical descriptions are provided in the Supplemental Information. No clear relationship was evident between the location of mutations and clinical phenotype. Familial ALS/FTD mutations in *CCNF* were present in the overall cohorts from these diverse geographic populations at frequencies ranging from 0.6 to 3.3%, which is comparable to the frequency of mutations in *TARDBP* (which encodes TDP-43) and *FUS* that have been reported in familial ALS cohorts^13^.

### Enrichment of rare protein-altering variants in sporadic ALS

To further evaluate whether rare protein-altering *CCNF* variants are associated with sporadic ALS, we examined an independent replication cohort of cases and controls. *CCNF* variants were identified in 611 Australian sporadic ALS cases and 1,424 cases from the ALS Data Browser data set. For the control data set, we used rare *CCNF* variants from the ExAC database (MAF <0.0001). Variants were subject to analysis if they were considered functional (nonsense, missense or frameshift) and passed quality filtres (described in Methods). Fisher's exact test revealed an enrichment of rare protein-altering variants in *CCNF* among sporadic ALS patients (1.39%) compared with controls (0.67%), *P*=6.58 × 10^−4^.

### UPS dysfunction and ubiquitination of TDP-43

*CCNF* (also called *FBXO1*) encodes the 786 amino-acid cyclin F protein (Fig. 1c). Cyclin F is a member of the cyclin protein family, but unlike most cyclins, it does not bind or activate a cyclin dependent kinase (CDK)^14^. Cyclin F is also a member of the F-box protein family characterized by an F-box motif that binds directly to SKP1, which in turn recruits CUL1 to form a SCF (SKP1-CUL1-F-box protein) E3 ubiquitin–protein ligase complex (SCF^Cyclin F^)^15^,^16^. E3s mediate the ubiquitination and proteasomal degradation of target proteins and are an integral component of the ubiquitin proteasome system (UPS).

**Figure 2:**
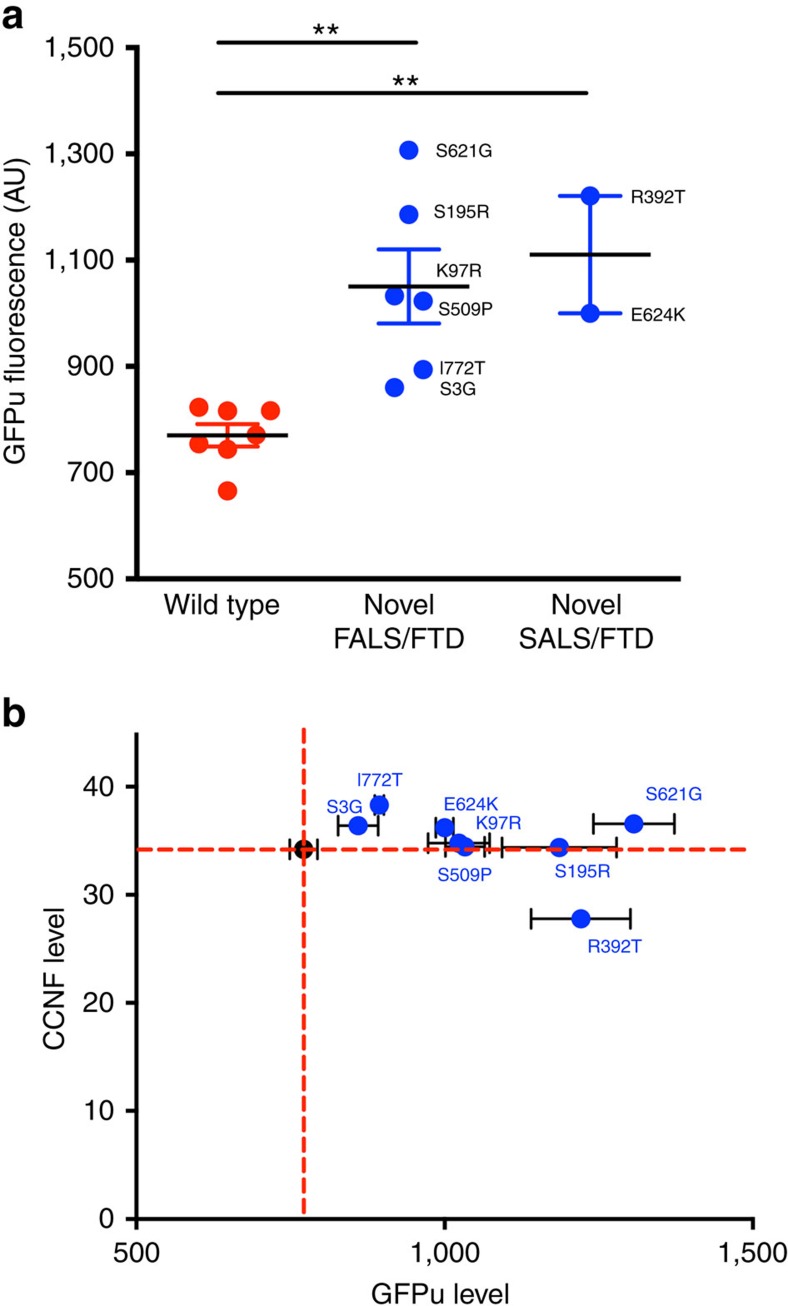
Mutant cyclin F impairs ubiquitin-mediated proteasomal degradation. NSC-34 cells were co-transfected with GFP^u^ and either wild type or mutant cyclin F, tagged with mCherry. GFP^u^ fluorescence intensity was analysed by flow cytometry 48 h post transfection. (**a**) Plot of GFP^u^ fluorescence intensity following flow cytometry. A significantly higher level of GFP^u^ fluorescence was observed in cells expressing novel cyclin F mutations (blue data points) when compared with those expressing wt *CCNF* (red data points) WT v FALS/FTD *P*=0.0017, d.f.=11; WT v SALS/FTD *P*=0.001, d.f.=7; two-tailed unpaired Student's *t*-test). (**b**) The higher level of GFP^u^ fluorescence was independent of the level of cyclin F as quantified using mCherry signal—R-squared=0.13. Red dashed lines represent the WT mean. Data are represented as mean,±s.e.m. *n*=3 (*n* is one experiment consisting of the mean of 50,000 cells); **P<0.01. d.f., degrees of freedom.

**Figure 3:**
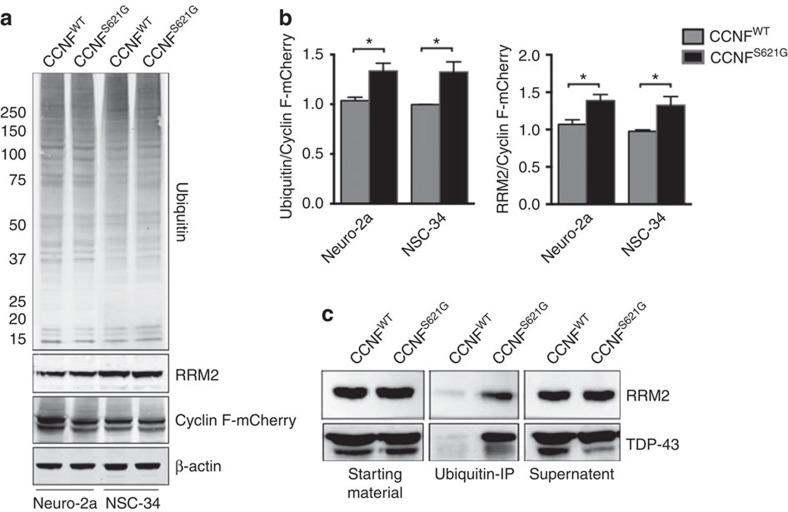
Increased ubiquitinated proteins in neuronal cell lines expressing mutant cyclin F. NSC-34 and Neuro-2a cells were transfected with wild-type or mutant cyclin F (p.S621G) expression constructs, and cells were collected after 24 h. (**a**,**b**) Cells transfected with mutant cyclin F (p.S621G) show increased levels (normalized to transfected cyclin F-mCherry) of ubiquitinated proteins and a known cyclin F–SCF complex target, RRM2 (Neuro-2a: Ubiq/cyclin F, *P*=0.025; RRM2/cyclin F, *P*=0.020. NSC-34: Ubiq/cyclin F, *P*=0.032; RRM2/cyclin F, *P*=0.023; two-tailed unpaired Student's *t*-test). (**c**) Immunoprecipitation (IP) of ubiquitinated proteins from transfected Neuro-2a cells (wild-type and mutant cyclin F (p.S621G)) show elevated levels of ubiquitinated RRM2 and TDP-43 in neuronal cells expressing mutant cyclin F. Full-length blots are presented in Supplementary Fig. 4. Data are represented as mean±s.e.m. *n*=3; *P<0.05.

Aberrant misfolded proteins are targeted for disposal by protein degradation pathways including the UPS and autophagic–lysosomal system, both of which are components of the complex network that maintains protein homeostasis (proteostasis). The accumulation of neuronal protein aggregates in ALS patients implicates dysfunction of the proteostasis network through inappropriate or inadequate response to aberrant proteins^17^. As a ubiquitin–protein ligase, cyclin F catalyses the transfer of activated ubiquitin to target proteins^15^. To investigate whether the ALS/FTD-associated variants in cyclin F lead to proteostasis dysfunction, we used the UPS reporter, GFP^u^, that consists of a 16 amino-acid degron (CL1, a specific substrate for the UPS), fused to the carboxyl terminus of green fluorescent protein (GFP)^18^. The degron sequence ensures rapid degradation of the GFP fusion through ubiquitin-mediated pathways, and the accumulation of this GFP reporter indicates UPS impairment. We confirmed that GFP^u^ signal correlates with UPS function in a motor neuron-like cell line (NSC-34) by inhibiting proteasome function using either the chemical inhibitor MG132, or expression of a mutant huntingtin exon 1 fragment containing an expanded polyQ sequence previously shown to inhibit the proteasome^19^ (Supplementary Fig. 2). Next, we co-transfected the NSC-34 cell line with GFP^u^ and either mutant cyclin F or wild-type cyclin F (Supplementary Table 4). Significantly higher levels of GFP^u^ fluorescence were observed for cyclin F with ALS/FTD-associated variants, indicating UPS dysfunction (Fig. 2a). This effect was independent of cyclin F expression levels (Fig. 2b). To examine whether the significant accumulation of GFP^u^ arose from the loss of proteasome activity or occurred upstream of the proteasome, we used two separate ubiquitin-independent small peptide 20S proteasome activity assays. These demonstrated that the UPS dysfunction was not due to altered proteolysis in the proteasome (Supplementary Fig. 3), consistent instead with the dysfunction stemming from abnormal ubiquitination or transport to the proteasome, mechanisms that are mediated, in part, by cyclin F. Consistent with these observations, western blotting confirmed the presence of significantly more ubiquitinated proteins in neuronal cell lines expressing mutant cyclin F (Fig. 3a,b). Collectively our data suggest that ALS/FTD-associated variants in *CCNF* modify the activity of SCF^Cyclin F^ resulting in overaccumulation of ubiquitinated proteins. To specifically demonstrate this, we found higher levels of the known SCF^Cyclin F^ target, RRM2 (Fig. 3a,b), including higher levels of ubiquitinated RRM2 (Fig. 3c), in neuronal cells expressing mutant cyclin F. Notably, we also observed substantially elevated levels of ubiquitinated TDP-43 in neuronal cells expressing mutant cyclin F (Fig. 3c).

## Discussion

We performed whole-genome linkage analysis and whole-exome sequencing in one of the largest ALS/FTD pedigrees to be described in recent years. ALS/FTD was significantly linked to a single locus encompassing *CCNF* and exome sequencing identified a single *CCNF* mutation that segregated with seven affected family members. We extended mutation discovery and rare variant analysis to ALS/FTD cohorts from diverse geographic populations, most of European ancestry. Analysis in a replication cohort showed a significant enrichment of novel and rare protein-altering *CCNF* variants in sporadic ALS patients. This suggests that aberrant cyclin F plays a role in both familial and sporadic ALS pathogenesis. Diagnosis of primary lateral sclerosis (PLS, an upper motor neuron disease) in a mutation carrier suggests that the spectrum of *CCNF*-linked motor neuron disease is wider than ALS.

Neuronal accumulations of ubiquitinated TDP-43 are a major pathological feature of almost all ALS cases, and the majority of FTD cases. However, the mechanisms responsible for TDP-43 ubiquitination are poorly understood. We demonstrated that ALS/FTD-associated variants in *CCNF* are responsible for abnormal increases in ubiquitination of TDP-43 and may to be responsible for wider changes in protein homeostasis. Further studies can now commence to confirm whether *CCNF* mutations lead to aberrant misfolded proteins and the accumulation of neuronal protein aggregates in ALS and FTD patients.

Abnormal protein homeostasis has been hypothesized to play a role in ALS pathogenesis. Convincing genetic linkage to familial ALS has also been shown for *UBQLN2*, which encodes ubiquilin 2, a protein that physically associates with ubiquitin ligases and proteasomes to mediate protein degradation^14^. Ubiquilin-2-positive neuronal inclusions are seen in affected motor neurons of some ALS and ALS/FTD patients, and ALS-linked mutations in ubiquilin 2 have also been shown to impair protein degradation mediated by the UPS^4^,^20^. Several other molecules that are functionally linked with cellular protein degradation pathways have also been associated with ALS (reviewed by Ling *et al*.^17^). Putative ALS-associated variants have been described in *SQSTM1*/p62, a ubiquitin binding protein with roles in protein degradation via the proteasome and autophagy. Interestingly, one of these *SQSTM1*/p62 variants and two cyclin F mutations described here, lie in a PEST sequence, a domain that is thought to act as a signal peptide for protein degradation^21^. ALS mutations have also been described in OPTN, an autophagic adaptor protein that binds substrates targeted for degradation and delivers them to autophagosomes^6^. OPTN-linked pathogenic mechanisms remain to be determined. A *VCP* mutation linked to inclusion body myopathy with Paget's disease of the bone and frontotemporal dementia (IBMPFD) impairs ER-associated degradation of ubiquitinated proteins from the ER^22^. *VCP* mutations have also been described in ALS cases but the mechanisms by which these lead to motor neuron degeneration remain unclear^10^. There is also evidence that defects in molecules functionally related to cyclin F play a role in other neurodegenerative diseases. Mutations in the related F-box only protein 7 gene (*FBXO7*) cause autosomal recessive, early-onset, parkinsonian-pyramidal syndrome and lead to decreased stability of the FBXO7 protein^23^. Similar to cyclin F, the F-box motif of FBXO7 interacts directly with Skp1 to form the SCF E3 ubiquitin–protein ligase complex^15^. Furthermore, around 10% of early-onset Parkinson's disease cases are caused by mutations in *PARK2*, which encodes Parkin, an E3-ubiquitin ligase. Parkin mutations impair degradation of its substrates, leading to accumulation of toxic products and eventually cell death^24^.

The known ALS proteins TDP-43 and FUS are capable of assembling into stress granules in response to oxidative stress and environmental insults, a process that is accelerated by ALS-linked mutations (reviewed by Li *et al*.^25^). Indeed, stress granules have been described as the crucibles of ALS pathogenesis^25^. Stress granules play a role in messenger RNA homeostasis and form during cellular stress, presumably to halt translation of non-essential transcripts. TIA-1, a messenger RNA-binding protein, translocates from the nucleus to seed stress granule formation in the cytoplasm. It was recently demonstrated that TIA-1 knockdown in mouse spinal cord and cerebellum led to marked and consistent increase in the expression of *CCNF*^26^.

Like most known ALS-linked molecules, it remains to be determined whether the functional consequences of *CCNF* mutations lead to a toxic gain of function or dominant-negative loss of function or haploinsufficiency. We found one *CCNF* frameshift variant (p.L372fs) that did not segregate with disease, suggesting that a dominant toxic gain of function may be required for mutant *CCNF* pathogenicity.

Segregation of the mutation with disease was clearly established in the large discovery family but could not be determined for other familial mutations in the discovery cohort due to the absence of DNA from other affected family members. This is now typical for familial ALS/FTD cohort studies, as almost all extended kindreds have been solved, leaving only single probands. We cannot conclusively determine that the additional novel familial *CCNF* mutations are the sole cause of disease, however, most substitute highly conserved residues and led to UPS dysfunction *in vitro*. Further resequencing of *CCNF* is warranted in other ALS and FTD cohorts, particularly those of European ancestry. Our data suggest that mutation of the encoded E3 ubiquitin ligase caused abnormal ubiquitination, implicating that common mechanisms, linked to protein homeostasis, underlie neurodegeneration in ALS and FTD. Additional functional studies, including animal models, can now commence to assess the pathogenic mechanism, and identify the targets of the mutant SCF^Cyclin F^ complex. These target molecules may, in turn, be considered candidate ALS and FTD genes.

## Methods

### Participants and samples

Patients, family members and unrelated controls were recruited under informed written consent as approved by the institutional review boards of Macquarie University, King's College London, Imperial College London, University of Massachusetts at Worcester, Stanford University, Emory University, McGill University Health Center, University of New South Wales, Mayo Clinic, Queen Elizabeth Hospital (South Birmingham Research Ethics Committee), Beaumont Hospital, Hospital Universitario 12 de Octubre, Fundación CIEN, IRCCS Istituto Auxologico Italiano and Fondazione IRCCS Istituto Neurologico ‘C. Besta', and the University of Tokyo. Patients were diagnosed with definite or probable ALS according to El Escorial criteria^27^. Patients had previously been screened for mutations/expansions in known ALS genes including *TARDBP*, *SOD1*, *FUS*, *UBQLN2*, *OPTN*, *VCP*, *PFN1* and *C9ORF72.* Formalin-fixed, paraffin-embedded cervical spinal cord sections from Australian patients and neurologically normal controls were provided by New South Wales Tissue Resource Centre (Sydney, Australia).

### Genetic linkage analysis

Genomic DNA was extracted from peripheral blood using standard protocols. For SNP-based genetic linkage analysis, genotyping was undertaken using the Affymetrix GeneChip Mapping 10Kv2.0 XbaI Array containing 10,204 SNP markers following the manufacturer's instructions and apparatus (Affymetrix, Santa Clara, CA, USA). The raw microarray feature intensities were processed using the Affymetrix Genotyping Tools software package (GCOS/GTYPE) to derive SNP genotypes, marker order and linear chromosomal location. Parametric multipoint linkage analysis of SNP data was performed using Merlin v1.1 with intermarker distances obtained from the Marshfield (http://research.marshfieldclinic.org/) and deCODE (http://www.decode.com/) sex-averaged SNP linkage maps.

For microsatellite-based genetic linkage analysis, genotyping was performed by deCODE genetics using 539 microsatellite markers spaced at an average of 8 cM. Two-point and multipoint linkage analysis was performed using the FASTLINK v4.1 program of the easyLINKAGE v5.08 package^28^,^29^ and graphed using R package lodplot v1.2. All available family members were used for analysis. Parameters for linkage analysis included autosomal dominant inheritance, age-dependent penetrance (0–30 years, 1%; 31–40 years, 30%; 41–50 years, 50%; 51–60 years, 70%; 61–70 years, 85%, >70 years, 90%), a disease allele frequency of 0.0001, equal male and female recombination, and equal marker allele frequencies. For fine mapping across the chr16p13.3 haplotype, control allele frequencies and, where available, CEPH allele frequencies were used to calculate two-point and multipoint LOD scores. Multipoint linkage analysis was performed under three penetrance models including the age-dependent penetrance classes described above, as well as 100 and 70% disease penetrance.

### Sequencing and mutation analysis

Exomes were captured using TruSeq Exome Enrichment kit or Agilent SureSelectXT Human All Exon V4. Paired-end sequencing was performed using the Illumina HiSeq2000 instrument.

*CCNF* exons were sequenced using Fluidigm Access Array target enrichment and the MiSeq sequencing platform (Illumina). Amplicon primers were designed using the Fluidigim D3 Design Studio to produce 150 bp amplicons targeting the exons of *CCNF*.

Validation and analysis of the *CCNF* mutations was performed by direct DNA sequencing following PCR amplification of coding exons (NM_001761). PCR products were Sanger sequenced using Big-Dye terminator sequencing and an ABI 3730XL DNA analyser (Applied Biosystems).

SNP genotyping in control individuals was performed using a custom TaqMan SNP genotyping assay according to the manufacturer's instructions (Life Technologies) and analysed using a Viia 7 real-time PCR system (Life Technologies).

Control exome data from 967 neurologically healthy individuals of predominantly Western European descent obtained from the Diamantina Institute, University of Queensland (Diamantina Australian Control Collection).

### Bioinformatics

Sequencing reads generated by the Illumina platform were aligned to the hg19 human genome assembly using BWA v0.6.1 (ref. 30), variants were called using SAMtools v0.1.16 (ref. 31) and annotated using ANNOVAR^32^. Annotated variants were compared among affected family members and controls using R v3.1.1 (http://www.R-project.org/)^33^. Filtering of variants was performed using dbSNP (releases 131, 132, 134 and 137; https://www.ncbi.nlm.nih.gov/SNP/), 1000 Genomes Project (Nov 2010 release; http://www.1000genomes.org/) and the ESP exome variant server (ESP6500 data release; http://evs.gs.washington.edu/EVS/).

Sequence reads generated by MiSeq were mapped to the hg19 human genome assembly using BWA^30^. GATK^34^,^35^,^36^ was applied to mapped reads for realigning and recalibration of base quality scores and variant calling. ANNOVAR^32^ was used for annotation of variants.

Quality filters for rare variant enrichment analysis include read depth ⩾10 in >75% of all samples and genotype quality >20.

Conservation of cyclin F orthologues was examined by aligning sequences from a variety of species (Entrez protein database; http://ncbi.nlm.nih.gov) using Clustal Omega (http://www.ebi.ac.uk/Tools/msa/clustalo/).

### Plasmids and cloning

Expression constructs comprising wild type and mutant *CCNF* cDNA fused with an N-terminal mCherry were developed using pmCherry-C1-*CCNF* (Addgene, https://www.addgene.org/32975/) and Q5 Site-Directed Mutagenesis kit (NEB) according to the manufacturer's protocol. All constructs were verified by DNA sequencing.

### Antibodies

The following primary antibodies were used in this study: rabbit polyclonal anti-cyclin F (cat # sc-952, Santa Cruz Biotechnology), mouse monoclonal anti-TDP-43 antibody (cat # H00023435-M01, Abnova), rabbit polyclonal anti-ubiquitin (Dako), mouse monoclonal anti-RRM2 (cat # ab57653, Abcam), anti-β actin (cat # A5441 [AC-15], Sigma-Aldrich). All antibodies were commercially sourced and validated according to the antibody data sheets.

### Cell lines

Mouse NSC-34 (neuroblastoma/motor neuron-enriched primary spinal cord hybrid) cells were provided by Prof Neil Cashman, University of Toronto. Mouse neuroblastoma Neuro-2a cells, and human neuroblastoma SH-SY5Y cells were from the ATCC repository (ATCC Product Nos. CCL-131 and CRL-2266).

### Confocal microscopy

Confocal fluorescence imaging was performed using a Leica DM6000 upright laser-scanning confocal microscope with Leica application suite advanced fluorescence software. Images were acquired with a × 63 (1.4 numerical aperture) oil-immersion objective. Images were acquired using sequential mode to avoid crosstalk between two dyes. Immunohistochemistry imaging was performed with a Zeiss Axio Imager 2 with ZEN pro program, using a × 40 objective.

### Cell culture and transfection for UPS assays

NSC-34 cells were maintained in DMEM (Sigma Aldrich) containing 100 U ml^−1^ penicillin, 100 mg ml^−1^ streptomycin and 10% (v/v) heat-inactivated fetal bovine serum (Sigma Aldrich). Cells were maintained in a humidified 37 °C incubator with 5% CO_2_. For the UPS assay and Enzo proteasome assay, NSC-34 cells were plated at a density of 50,000 cells per well in six-well plate. For the Abcam proteasome assay, NSC-34 cells were plated at a density of 2,000 cells per well in 96-plate well plate. Transfections were carried out using Lipofectamine LTX (Life Technologies) according to manufacturer's protocol. Transfections included 2.5 μg DNA for the UPS assay, 5 μg DNA for the Enzo proteasome assay or 0.1 μg DNA for the Abcam proteasome assay.

### Ubiquitinated protein immunoprecipitation

Co-transfected Neuro-2a cells were lysed and total protein was extracted with sonication (10 s, Setting 3, Branson Sonifier 450) in extraction buffer (1% (v/v) Nonidet P-40 in TBS (50 mM Tris-HCl, pH 7.5, 150 mM NaCl) with protease inhibitor cocktail). Cellular debris was pelleted at 18,000*g* (30 min at 4 °C). Protein concentration was estimated using the BCA Protein Assay Reagent (Pierce Biotechnology). Typically, 3 μg of anti-ubiquitin (Dako) per 500 μg of protein extract was used for immunoprecipitations. Protein A/G magnetic beads (Pierce Biotechnology) were used to capture the antibody:protein complex. Immunoprecipitates were washed with TBS+1% (v/v) NP-40 (3 × ) to remove non-specifically bound proteins, and then resuspended in 1 × LDS buffer with 50 mM DTT, and heated at 95 °C for 10 min.

### UPS reporter assay and 20S proteasome activity assay

For the UPS reporter assay, NSC-34 cells were cultured in six-well plates for 24 h, followed by co-transfection with a UPS-specific degron GFP^u^ and cyclin F constructs. The GFP^u^ reporter contains a CL1 sequence which signals ubiquitination and degradation by the proteasome^18^,^37^. The resulting GFP signal is a reporter for rate of protein degradation in the cell. Cells were collected 48 h post transfection by trypsin and resuspended in PBS (Sigma-Aldrich). In order to show the sensitivity of GFP^u^ construct, cells were either transfected with GFP^u^ for 24 h followed by MG132 (Calbiochem) treatment at various doses for a further 24 h or co-transfected with GFPu and mCherry with or without MG132, or lastly co-transfected with GFP^u^ and either Httex125Q or Httex146Q and incubated for 48 h. The fluorescent intensity GFP in collected cells was analysed using flow cytometry Becton Dickinson LSR II. At least 50,000 cells per treatment were collected. Data were gated on mCherry-positive cells with an excitation at 541 nm and an emission at 575 nm. The geometric mean of the GFP signal from this population was collected with an excitation at 488 nm and an emission at 520 nm. Data were from three independent experiments.

The proteasome enzymatic activity of cyclin F was measured using either a 20S proteasome assay kit from Enzo Life Sciences or Abcam following the manufacturer's instructions. For the Enzo Life Sciences proteasome activity kit, NSC-34 cells were transfected wild-type pmCherry-C1-CCNF or mutant pmCherry-C1-CCNF in six-well plates and protein extract was generated by freeze–thaw lysis 48 h post-transfection. Protein concentration was measured by BCA protein assay (Thermo Scientific) and equal amount of protein was used in the assay. The proteasome activity was measured by hydrolysis of a fluorogenic peptide substrate Suc–Leu–Leu–Val–Tyr–AMC (AMC: 7-amino-4-methylcoumarin). The substrate is cleaved by the 20S proteasome and the release of free AMC fluorophore is used as an indication of proteolytic activity. The fluorescent signal was measured by FLUOstar OPTIMA fluorescence plate reader (BMG Labtech) with an excitation at 360 nm and an emission at 460 nm. The data were collected at 2-min interval for 50 min. For the Abcam proteasome activity kit, NSC-34 cells were transfected with wild-type pmCherry-C1-CCNF or mutant pmCherry-C1-CCNF in 96-well plates for 48 h. A proteasome substrate Leu–Leu–Val–Tyr–R110 was added directly to the cells and incubated at 37 °C for 1 h. The substrate is cleaved by the 20S proteasome and the fluorescent signal generated from the cleavage is used as an indication of proteolytic activity. The fluorescent signal was measured by FLUOstar OPTIMA fluorescence plate reader (BMG Labtech) with an excitation at 490 nm and an emission at 525 nm.

### Statistical analysis of *in vitro* assays

All statistical analyses were performed using GraphPad Prism Software. Two-tail unpaired Student's *t*-tests (*P*<0.05) were used for grouped GFP^u^ data (wild-type, cyclin F mutation (familial and sporadic)). One-way analysis of variance with Dunnett's multiple comparison tests were used for comparison of GFPu levels in the presence of cyclin F variants, with wild type. Statistical analyses of other *in vitro* assays were performed using two-tailed unpaired Student's *t*-tests (*P*<0.05). All values were mean±s.e.m. The data met the assumptions of each specific statistical test. Variance was similar between groups. Sample size was chosen based on results from pilot studies.

## Additional information

**How to cite this article:** Williams, K. L. *et al*. *CCNF* mutations in amyotrophic lateral sclerosis and frontotemporal dementia. *Nat. Commun.* 7:11253 doi: 10.1038/ncomms11253 (2016).

## Supplementary Material

Supplementary Information Supplementary Figures 1-4, Supplementary Tables 1-4 and Supplementary Note 1

## Figures and Tables

**Table 1:** ALS and/or FTD mutations in *CCNF*.

**Amino-acid change**	**Nucleotide change**	**Exon**	**Cohort**	**Control samples (Sanger and exome)**	**Public database MAF**
*Familial*					
p.S3G	7A>G	1	1/99 US FTLD-ALS	0/1038 US controls0/657 AU controls0/967 AU control exomes	Absent
p.K97R	290A>G	4	1/159 UK FALS	0/897 UK controls0/967 AU control exomes	Absent
p.S195R	585T>G	6	1/30 SP FALS	0/967 AU control exomes	Absent
p.S509P	1525T>C	13	1/99 IT FALS1/168 CA SALS	0/361 CA controls0/967 AU control exomes	Absent
p.S621G	1861A>G	16	1/75 AU FALS	0/864 AU Sanger controls0/967 AU control exomes	Absent*
p.I772T	2315T>C	17	1/159 UK FALS	0/897 UK controls0/967 AU control exomes	Absent
					
*Sporadic*					
p.T181I	542C>T	6	1/283 JA SALS	0/514 JA controls0/967 AU control exomes	Absent*
p.R392T	1175G>C	11	1/99 US FTLD	0/1038 US controls0/967 AU control exomes	Absent
p.T543I	1628C>T	15	1/283 JA SALS	0/514 JA controls0/967 AU control exomes	Absent
p.E624K	1870G>A	16	1/49 US SALS trios	0/801 AU Sanger controls0/967 AU control exomes	Absent*

ALS, Amyotrophic lateral sclerosis; AU, Australian; CA, Canadian; ESP, Exome Sequencing Project; IT, Italian; MAF, minor allele frequency; SP, Spanish; UK, United Kingdom; US, USA.

*CCNF* accession NM_001761. Data were mined from whole exome or genome sequence data and validated by Sanger sequencing. Public databases include dbSNP; NHLBI ESP Exome Variant Server and 1000 Genomes Project.

^*^Variant was present as a singleton in ExAC, frequency of 8.6 × 10^−6^.
