# Supplementary material for: CCNF mutations in amyotrophic lateral sclerosis and frontotemporal dementia
Source: Nat Commun. 2016 Apr 15;7:11253. doi: 10.1038/ncomms11253 (PMC4835537; doi:10.1038/ncomms11253)
Supplement: Supplementary Information — Supplementary Figures 1-4, Supplementary Tables 1-4 and Supplementary Note 1 [file ncomms11253-s1.pdf]

SUPPLEMENTARY INFORMATION

Supplementary Figures

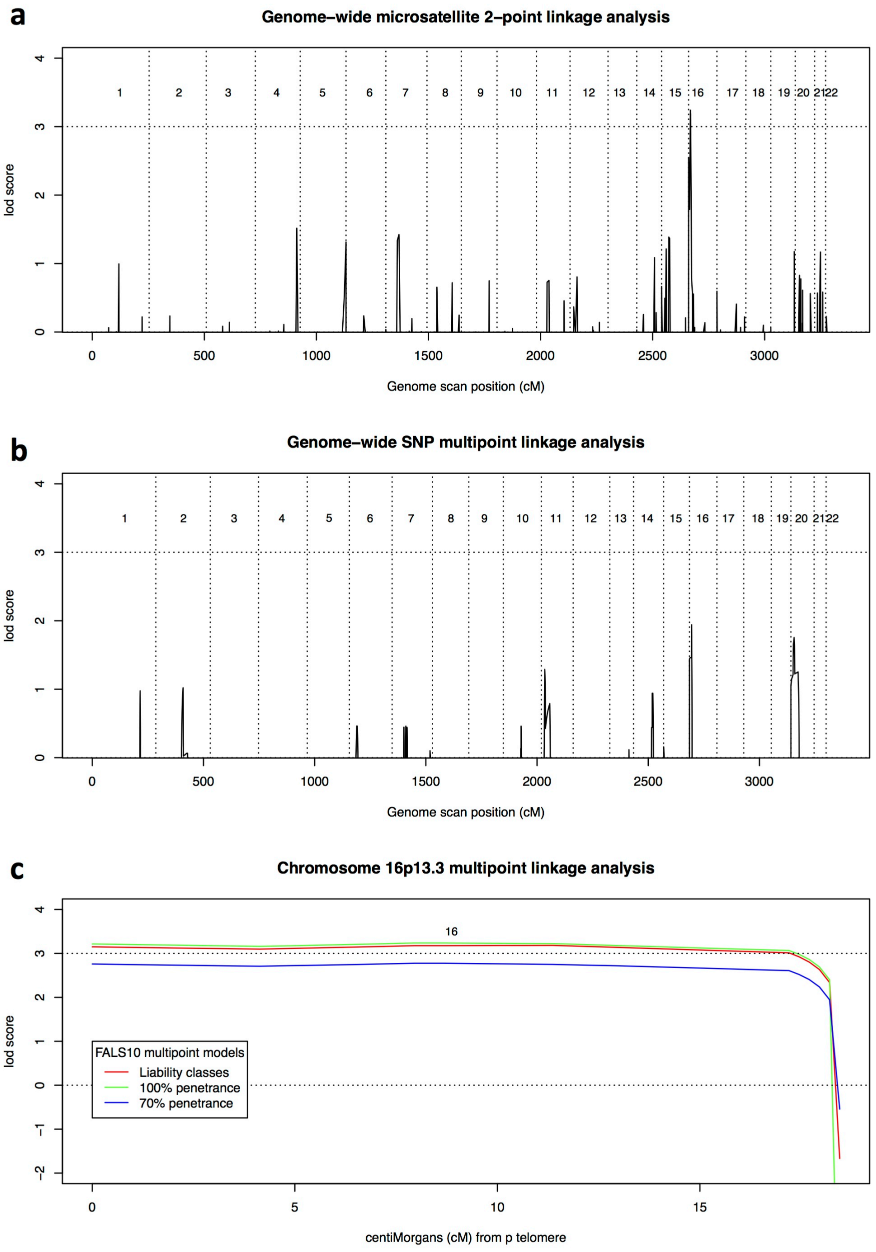

Supplementary Figure 1. Summary of genetic linkage data from analysis of family FALS10. Summary of genome-wide linkage analysis using microsatellite markers **(a)** and SNPs **(b)**. Black vertical lines indicate positive LOD scores. No X-chromosome marker generated a LOD score > 0, hence they are not shown. **(c)** Multipoint linkage analysis of the linked interval on chr16p13.3, using three disease penetrance models.

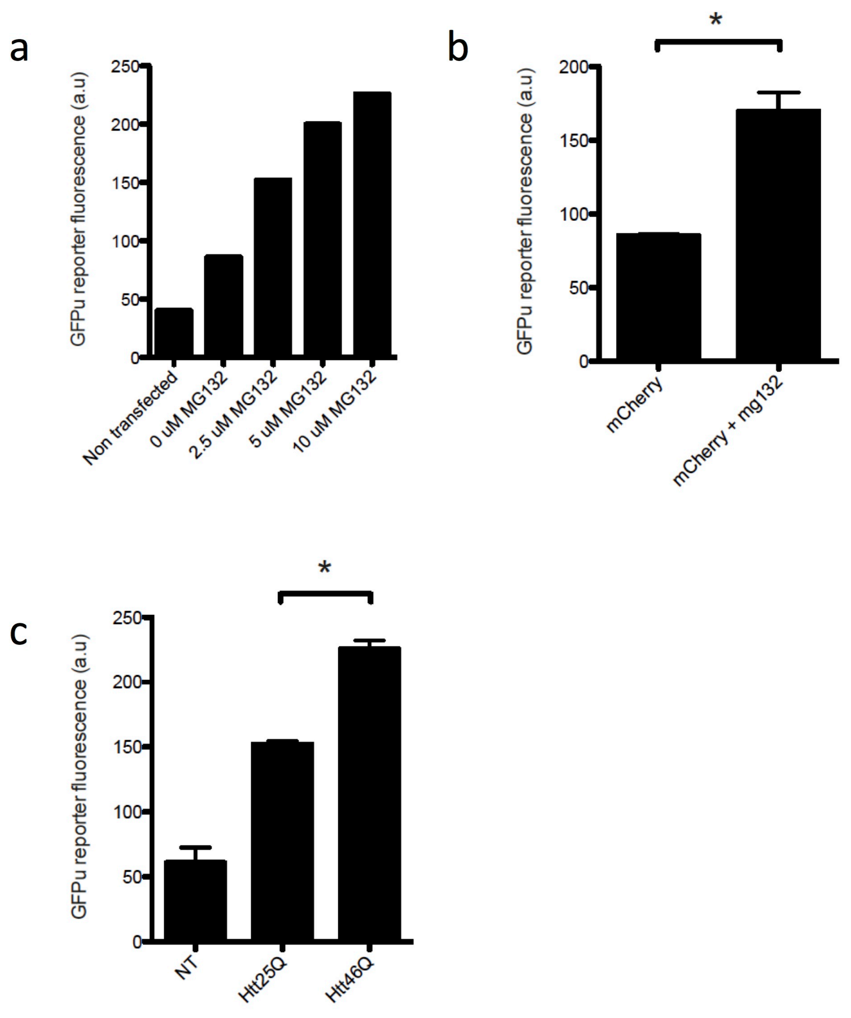

Supplementary Figure 2. GFP^{u} signal correlates with UPS function. NSC-34 cells were transfected with GFP^{u}. The fluorescence intensity of GFP^{u} was analyzed with flow cytometry. **(a)** Cells were treated with various doses of MG132 at 24 h post-transfection and incubated for a further 24 h before analysis. A dose-dependent increase of GFP^{u} fluorescence was observed when proteasome function was inhibited by MG132. **(b)** NSC-34 cells were co-transfected with GFP^{u} and empty pmCherry vector. After 24 h, cells were treated with 5ug/ml MG132 and incubated for a further 24 h before analysis. A significant accumulation of GFP^{u} fluorescence was found ($p = 0.003$, $df = 4$), indicating that co-transfection with pmCherry did not affect proteostasis of GFP^{u}. **(c)** NSC-34 cells were co-transfected with Huntingtin exon1 fragments containing an expanded polyQ (Htt25Q and Htt46Q). The fluorescence was measured 48 h post-transfection. Htt25Q and Htt46Q have been previously shown to inhibit the proteasome. A significant increase of GFP^{u} fluorescence was observed ($p = 0.0005$, $df = 4$). Data are represented as mean ± s.e.m. n = 3; $*P < 0.05$.

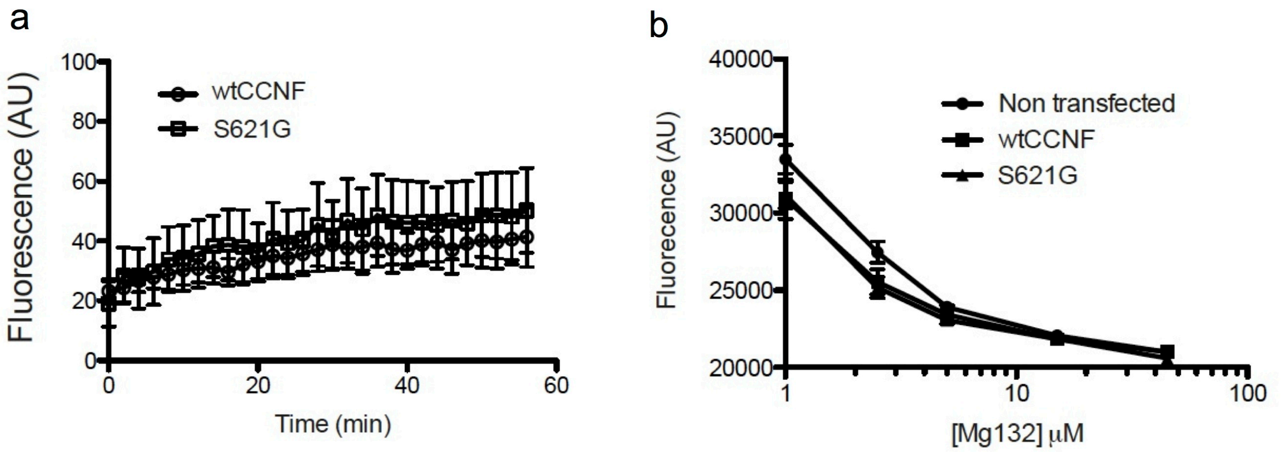

Supplementary Figure 3. Dysfunction of ubiquitin-mediated degradation was not due to altered proteasome activity. NSC-34 cells were transfected with either wild type or mutant *CCNF* tagged with mCherry. Proteasome enzymatic activity was measured by fluorogenic proteasome assays 48 h post-transfection. **(a)** Protein lysate was generated from transfected NSC-34 cells and the kinetics of 20S proteasome chymotrypsin-like protease activity was measured using the Suc-LLVY-AMC fluorogenic peptide substrate (Enzo Life Sciences). Fluorescence changes were measured at 2 min intervals for 50 min. No significant difference was found between wild type and mutant cyclin F. **(b)** Proteasome activity was measured in intact NSC-34 cells using a cell permeable proteasome substrate (Abcam). No significance difference was found between wild type and mutant cyclin F. Data are represented as mean ± s.e.m. n = 3.

Fig. 3a

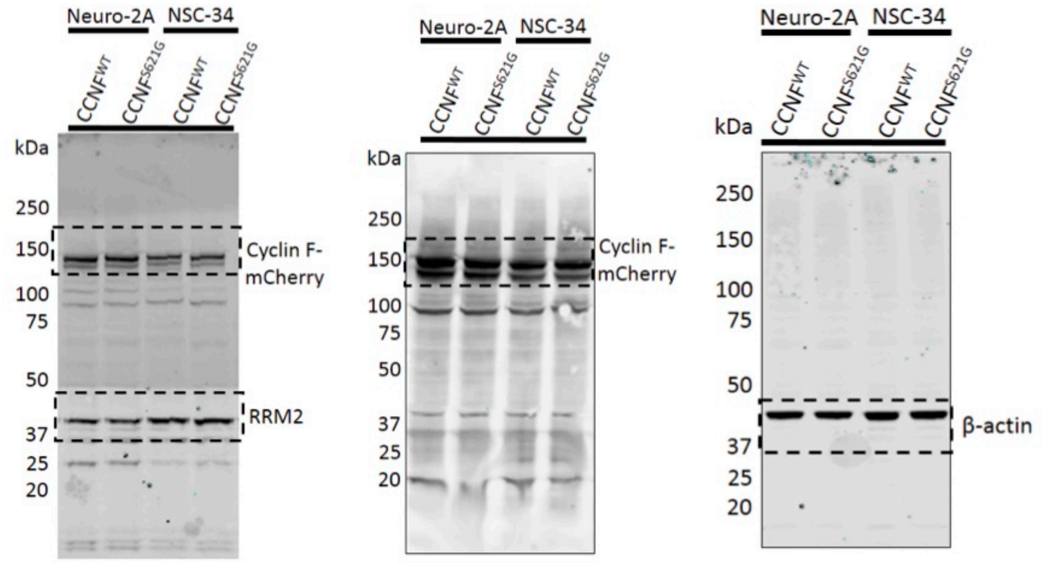

Fig. 3c

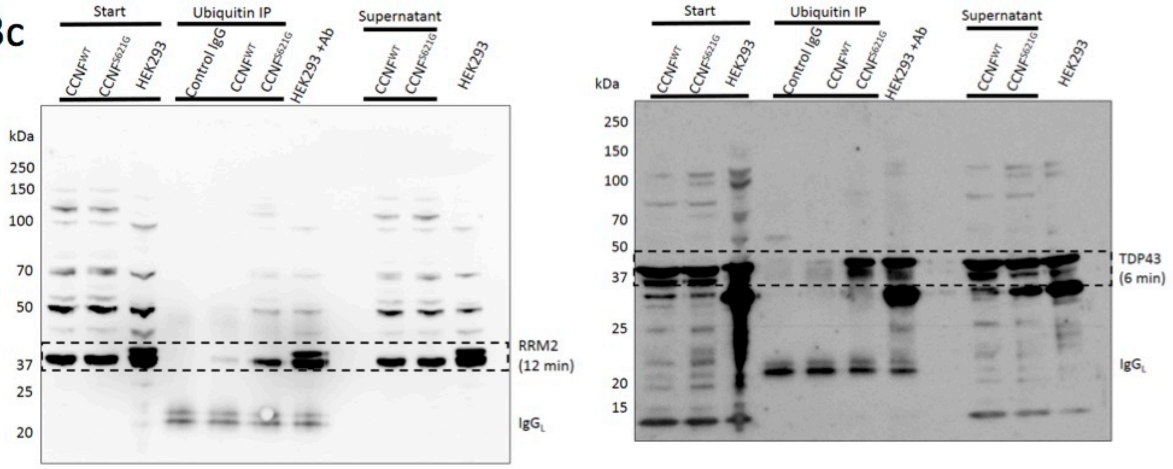

Supplementary Figure 4. Full-length pictures of blots presented in Figure 3.

Supplementary Tables

Supplementary Table 1. Two-point LOD scores for family FALS10 using microsatellite markers from the chromosome 16 minimum candidate disease haplotype.

**Marker**	**cM**	**LOD (theta=0)**	**Allele size**	**Allele frequency**
D16S521	1.14	1.72	166*	0.017857*
D16S3401	1.143	2.55	165	0.176471
D16S3024	5.27	1.79	228	0.3125
**D16S3082**	**9.09**	**3.24**	**208***	**0.160714***
D16S475	9.83	2.89	180	0.1875
D16S2622	9.831	2.48	79*	0.185185*
**D16S3065**	**9.832**	**3.19**	**162***	**0.017857***
D16S3134	12.51	2.06	161*	0.42*
D16S423	14.05	0.79	125*	0.357143*
D16S3088	18.34	0.57	219*	0.462963*
D16S418	19.59	-3.23	179	0.0625

LOD scores >3 are in bold. *CEPH allele size and frequency.

Supplementary Table 2. Filtering steps to identify causative gene mutation in ALS/FTD family FALS10.

Filter	Variants
Total variants identified	292,989
Present in all affected	35,930
Alters amino acid sequence	5,109
Novel variants	2 (dbSNP, EVS, 1000 genomes, ExAC)
Within linkage region chr16p13.3*	1

* No novel variants were identified on chromosomes 11 or 20.

Supplementary Table 3. Characteristics of familial ALS/FTD patients with *CCNF* mutations.

Family	Amino acid change	Origin	Diagnosis	Sex	Age of onset (years)	Duration (months)	Site of Onset	Cognitive impairment
FALS10; III:1	S621G	Australia	ALS	M	54	12	Bulbar	
FALS10; II:14	S621G	Australia	ALS	F	61	60	Limb	
FALS10; II:13	S621G	Australia	ALS	F	Died at 81	unknown	Bulbar	
FALS10; II:10	S621G	Australia	FTD	F	Died at 72	unknown		FTD
FALS10; III:15	S621G	Australia	ALS	M	58	>6	Limb	
FALS10; III:4*	S621G	Australia	ALS	M	42	44	Limb	
FALS10; II:2*	S621G	Australia	ALS	F	Died at 74	unknown	unknown	Multiple strokes, dementia
FALS10; II:6*	S621G	Australia	ALS	M	Died at 68	unknown	Limb	
NA09-350	S3G	USA	FTLD-ALS	M	66	14	unknown	FTD
ALS0057	K97R	UK	ALS	F	44	>20	Limb	
ALS0084	I772T	UK	PLS	F	62	unknown	Limb	
SP-37	S195R	Spain	ALS	F	54	21	unknown	
IT	S509P	Italian	ALS	M	57	unknown	Limb	

*Mutation could not be confirmed through sequencing as no DNA was available, however segregation analysis confirmed the presence of the mutation.

Supplementary Table 4. Novel and rare protein-altering *CCNF* variants identified in the ALS and/or FTD discovery cohorts.

Amino acid change	Nucleotide change	rs ID	ALS/FTD MAF (population)	Control MAF (n>950)	Public database MAF	ExAC Frequency (population specific)
p.H69Y[#]	205C>T	Novel	0.002976 (CA SALS)	0	Absent	-
p.A74T	220G>A	rs4589553	0.006667 (AU FALS)	0	NA (dbSNP)	-
p.G161R	481G>A	rs148159882	0.019231 (US SALS)	0	NA (dbSNP)<br>0.000077 (ESP)	0.000062 (European, non-Finnish)
p.T181I	542C>T	Novel	0.003534 (JA SALS)	0	Absent	0.000116 (East Asian)
p.V318G	953T>G	rs201540325	0.006667 (AU FALS)	0.074744	NA (dbSNP)	-
p.R344K	1031G>A	rs371050277	0.015152 (JA FALS)	0	NA (dbSNP)<br>0.000077 (ESP)	0.000695 (East Asian)
p.L372fs[§]	1114_1115insC	Novel	0.006667 (AU FALS)	0	Absent	-
p.R392T	1175G>C	Novel	0.010101 (US FTLD)	0	Absent	-
p.R406Q	1217G>A	rs146438723	0.005051 (US FTLD-MND)	0.002068	NA (dbSNP)<br>0.001616 (ESP)	-
p.E528Q	1582G>C	Novel	0.001767 (JA SALS)	0	0.002743 (HGVD)	0.000463 (East Asian)
p.L531R	1592T>C	rs372723774	0.003155 (JA FALS & JA SALS)	0	NA (dbSNP)<br>0.001667 (HGVD)	0.003704 (East Asian)
p.T543I	1628C>T	Novel	0.003534 (JA SALS)	0	Absent	-
p.R574Q	1721G>A	rs369730776	0.002976 (CA SALS)	0	NA (dbSNP)<br>0.000231 (ESP)	0.000018 (European, non-Finnish)
p.F604I	1810T>A	rs118131564	0.012367 (JA SALS)	0.004785	0.000693 (ESP)<br>0.020202 (1000 genomes)<br>0.014602 (HGVD)	0.011661 (East Asian)
p.E624K	1870G>A	Novel	0.020408 (US SALS trios)	0	Absent	0.000016 (European, non-Finnish)
p.Q669*	2005C>T	rs145521789	0.005051 (US FTLD-MND)	0	NA (dbSNP)<br>0.000077 (ESP)	0.000015 (European, non-Finnish)
p.R691Q	2072G>A	rs148419125	0.001767 (JA SALS)	0	0.000308 (ESP)<br>0.000459 (1000 genomes)<br>0.002060 (HGVD)	0.001970 (East Asian)
p.T698M	2093C>T	rs201460257	0.006944 (AU sporadic FTD)	0	0.000 (dbSNP)<br>0.000459 (1000 genomes)	-
p.V714M	2140G>A	rs61755288	0.017606 (AU FALS & CA SALS)	0.013220	0.010619 (ESP)<br>0.005510 (1000 genomes)	0.014407 (European, non-Finnish)

CCNF accession NM_001761. AU, Australian; CA, Canadian; JA, Japanese; UK, United Kingdom; US, USA; HGVD, Human Genetic Variation Database (Japanese; http://www.genome.med.kyoto-u.ac.jp/SnpDB); NA, not available. [#] H69Y is listed here because other variants at the H69 codon are present in dbSNP, (including p.H69D, rs369245530). [§] Variant does not segregate with disease.

Supplemental notes

Additional clinical information for familial ALS/FTD cases with *CCNF* mutations.

Family FALS10 In the linked ALS/FTD family FALS10, the average age of onset was 53.8 ± 8.3 years (range 42 – 61) and the average duration of disease was 38.7 ± 24.4 months (range 12 – 60 months). Including age information from four at-risk carriers, the age-dependent penetrance in family FALS10 is estimated to be 50% by age 56 and 100% by 61.

FALS10, III:4 The patient started to show symptoms of ALS at age 42 with weakness of movement of right thumb, tiredness, weakness of legs, twitches in muscles, cramping of calves and slurring of speech. No swallowing or breathing difficulty was observed. Examination revealed a mild slurring. There was mild weakness of the palate and pharyngeal muscles. Upper limb weakness was confined to the hands. Wasting was present in the right thenar region and in the abductor digiti minimi. Also weak were the adductor pollicis on the right and the extensor pollicis longus and brevis on both sides, but more on the right. In the lower limbs weakness was confined to the muscles below the knee with the left side more affected. Tibialis anterior, toe extensors and the peronei were considerably affected on both sides. The right tibialis posterior was only mildly affected. No obvious wasting was present in the lower limbs. Widespread occasional fasciculations were noted in all limbs. Sensory examination was normal.

FALS10, II:13 This patient presented with a history of progressive dysarthria with weakness in the arms and legs for several months. Upon examination, the patient had dysarthria, but no tongue fasciculations or weakness. The jaw jerk was present and brisk and cranial nerves were intact. There was some reduction of facial expressions and bradykinesia with mild increased tone in the neck and extremities. The patient had grade 4/5 strength (MRC grading) for shoulder abduction and small muscles of the hands. Proximal leg weakness was also observed. Fasciculation was notes generally in the upper limbs and the right quadriceps. Ankle tendon reflexes were reduced. EMG showed wide spread denervation including the tongue. Nerve conduction studies did not indicate a neuropathy. No dementia is present, but the patient has mild parkinsonian features.

FALS10, III:1 The first symptom observed in this patient was the inability to move the tongue properly. Upon examination, the patient had localized tongue fasciculations that were confirmed by EMG and nerve conduction studies. No other fasciculations were evident, however the patient had extensor planters. There was no evidence of nerve conduction failure. During disease progression, the patient had significant speech problems and fasciculation were evident in the arms and legs.

FALS10, II:4 This patient had asymmetrical muscular atrophy more marked in the right upper limb progressing to the left upper limb. There was bilateral weakness of dorsiflexion of the feet. The disease progressed distally rather than proximally with no convincing upper motor neurone features.

SP-37 Age at onset was 54.3 years with a duration of illness about 20.5 months. Her father had dementia plus parkinsonism. Her grandmother had generalized weakness (dysphagia and dysarthria) and died at age 75.

IT Male, onset at age 57 in his left leg, classic ALS with UMN and LMN signs, no dementia. His mother and a sister of his maternal grandmother had ALS; his maternal grandmother had dementia, while a maternal aunt had PD.

NA09-350 Male, Caucasian. Clinical and pathological diagnosis of FTLD-MND with TDP-43 type 1 pathology. Age at onset was 66.3 years, age at death was 67.5 years. Balance disturbances with falls and personality change (angry, irritable, apathetic, withdrawn). Re: family history of dementia in first degree relative, relevant for mother with Alzheimer disease diagnosed when she was 76 years of age, passing away when she was 86 years of age.
